# Regional Resource Assessment During the COVID-19 Pandemic in Italy: Modeling Study

**DOI:** 10.2196/18933

**Published:** 2021-03-09

**Authors:** Pietro H Guzzi, Giuseppe Tradigo, Pierangelo Veltri

**Affiliations:** 1 Department of Surgical and Medical Sciences University of Catanzaro CZ Italy; 2 Ecampus University Novedrate Italy

**Keywords:** COVID-19, data analysis, ICU, management, intensive care unit, pandemic, outbreak, infectious disease, resource, planning

## Abstract

**Background:**

COVID-19 has been declared a worldwide emergency and a pandemic by the World Health Organization. It started in China in December 2019, and it rapidly spread throughout Italy, which was the most affected country after China. The pandemic affected all countries with similarly negative effects on the population and health care structures.

**Objective:**

The evolution of the COVID-19 infections and the way such a phenomenon can be characterized in terms of resources and planning has to be considered. One of the most critical resources has been intensive care units (ICUs) with respect to the infection trend and critical hospitalization.

**Methods:**

We propose a model to estimate the needed number of places in ICUs during the most acute phase of the infection. We also define a scalable geographic model to plan emergency and future management of patients with COVID-19 by planning their reallocation in health structures of other regions.

**Results:**

We applied and assessed the prediction method both at the national and regional levels. ICU bed prediction was tested with respect to real data provided by the Italian government. We showed that our model is able to predict, with a reliable error in terms of resource complexity, estimation parameters used in health care structures. In addition, the proposed method is scalable at different geographic levels. This is relevant for pandemics such as COVID-19, which has shown different case incidences even among northern and southern Italian regions.

**Conclusions:**

Our contribution can be useful for decision makers to plan resources to guarantee patient management, but it can also be considered as a reference model for potential upcoming waves of COVID-19 and similar emergency situations.

## Introduction

COVID-19 is a disease that was reported in Wuhan, China in December 2019 [1]. It has been stressing health structures and governments worldwide due to the difficulties in containing its diffusion [2-4]. The virus appears as a flu, but it attacks the pulmonary apparatus, and on average, 6% of symptomatic patients require hospitalization in intensive care units (ICUs) due to severe respiratory syndromes. The rapid diffusion of the virus caused a high number of infections. Only a fraction of patients who are infected need hospitalization in ICUs, a relatively high number of which do not survive the virus. In Italy, in almost 2 months during the first peak, there have been more than 100,000 known infections and nearly 35,000 COVID-19–related deaths [5-7].

The virus also spread in other countries across the world with different modalities and aggressiveness. During its first wave, from March to May 2020, Italy had the second highest number of patients who were infected. All countries reported difficulties in answering to the high number of requests for ICU beds and reliable detection of the real infection numbers. In countries where the virus spread later with respect to Italy, such as the United States, France, Spain, and other European countries, it diffused with similar trends but with different absolute numbers, as reported by the World Health Organization [3,8-13]. Nevertheless, the impact on health structure resources has been similar. In fact, the number of infections detected is strictly related to the number of swab tests performed in the population. Tradigo et al [14] assessed the real number of people who are infected with respect to the known ones. In contrast, the number of infections is much higher than the known ones and includes patients who are asymptomatic (ie, those who got the infection but who did not manifest any symptoms). The number of ICU beds is always related to the number of real infections.

Regions such as Lombardia in the north of Italy have been strongly affected by COVID-19, and it seems that this is due to a large number of patients who were asymptomatic already in the middle of January 2020 and late adoption of containment measures such as limitation of circulation and delay in applying rules such as smart working [15-17].

We focus on the problem of rapidly estimating resources during the exponential phase of the COVID-19 emergency, in particular, being aware of the differences among regions in terms of health structure resources such as ICU beds. We show how the proposed model scales at the regional level and how it can help decision makers plan expansions of resources near saturation or reroute patients to neighboring regions.

The prediction of COVID-19 diffusion is a relevant problem, and it has been discussed in other papers [18-20]. Some papers do not consider the regional level and local differences that are relevant in some countries such as Italy. For instance, Li et al [21] developed a model starting from Hubei Province data, and they used the model for prediction in other countries such as Italy and Korea. Other papers did not consider the prediction of ICU resources. Conversely, our study is scalable on a regional level, and it is able to predict ICU needs. In a previous study [22-24], we developed a preliminary model for resource planning; here, we present an extension of the model with a particular focus on the assessment of the predictions.

The model presented here has been assessed comparing the simulated and predicted resource values with the measured ones. The rapid diffusion trend in high-income countries’ populations and in cities with high density stressed the health structure in many countries. Indeed, a small portion of patients with COVID-19 require ICU admission. The exponential diffusion in terms of an increased number of infections per day required a larger number of ICU beds than the ones available. We report our model as being scalable at both the regional and subregional levels. We claim that it can be used in different countries and in future contexts where virus diffusion will require well-planned health resource management [13,25].

The paper is structured as follows. The Methods section reports the proposed assessed model and the Italian infection data. The Results section reports the application of the model on three sample regions out of 20, Lombardia (north), Toscana (central), and Sicilia (south). The Discussion section reports on the limitations of this study and comparisons with other work. However, our model is general enough to be successfully applied to other pandemic situations in other contexts.

## Methods

### ICU Situation in Italy

Italy was affected by COVID-19 by the end of January 2020, starting from northern regions such as Lombardia and Veneto. By the end of February, the increasing trend of infection numbers per day obliged the governments at the regional level—and at the national level starting on March 10–to introduce containment measures.

For example, on March 26, 2020, in Italy, we had 24,747 total reported COVID-19 infection cases, of which 20,603 had the disease, 1809 had died, and 2335 had recovered from it. Regarding patients who were infected, 9268 were treated in their homes since they did not have severe illness, 9663 were hospitalized, and 1672 were admitted to ICUs. The trend continued increasing until April 19, 2020, which has been the peak of COVID-19 infections in Italy.

In reaction to the exponential growth of patients who are infected that require hospitalization, one possible measure adopted by many countries has been to build emergency hospitals dedicated to patients with COVID. In Italy, one strategy consisted in improving existing structures by extending the number of ICU resources and beds, and using dedicated health structures. For example, one study [26] focuses on accelerating the process of acquiring and furnishing hospitals with assisted breathing devices.

Italy has approximately 5200 ICU beds in total, which have been dimensioned by design to be equal to 80% of their average occupancy at any given time. In addition, they are allocated at a regional level proportional to the local population and are usually managed locally. Table 1 reports the ICU bed distribution among regions associated with the demography. The COVID-19 pandemic called these choices into question, thus introducing the necessity of emergency units in cities where the virus rapidly diffused and where existing resources were limited.

**Table 1:** Distribution of ICU beds in each Italian region ordered by regional population. The number of beds could increase in the future due to government investments for the emergency.

Region	ICU^a^ beds, n	Population, n	ICU beds per citizen (%)
Lombardia	1067	10,060,574	0.0106
Lazio	590	5,879,082	0.0100
Campania	350	5,801,692	0.0060
Sicilia	346	4,999,891	0.0069
Veneto	498	4,905,854	0.0102
Emilia Romagna	539	4,459,477	0.0121
Piemonte	320	4,356,406	0.0073
Puglia	210	4,029,053	0.0052
Toscana	450	3,729,641	0.0121
Calabria	110	1,947,131	0.0056
Sardegna	150	1,639,591	0.0091
Liguria	70	1,550,640	0.0045
Marche	108	1,525,271	0.0071
Abruzzo	73	1,311,580	0.0056
Friuli Venezia Giulia	80	1,215,220	0.0066
Trentino Alto Adige	71	1,072,276	0.0066
Umbria	30	882,015	0.0034
Basilicata	49	562,869	0.0087
Molise	30	305,617	0.0098
Valle D’Aosta	15	125,666	0.0119
Italy (total)	5156	60,359,546	0.0085

^a^ICU: intensive care unit.

Because of ICU bed limitations, many patients have been moved from ICUs to subintensive units or to other regions to free up spaces. Indeed, ICU slots are often used for treating postsurgery patients and patients affected by pulmonary diseases. At the date of the peak (ie, April 19, 2020), almost 2635 ICU beds were occupied, 108,257 infections were confirmed by swab tests, and 25,033 patients had recovered without using ICU beds. Thus, most of the infections were asymptomatic, and patients quarantined at home. Even if the number of required ICU beds is less than the total number of available ICU beds in Italy (see Table 1), the infection distribution is not homogeneous among regional departments and does not follow a regular geographical distribution.

Thus, performing a flexible and reliable model that can predict and control resource requirements and distribution at a regional scale is required. The number of patients in the ICUs is also related to the requests of other clinical units such as emergency units for non–COVID-19, but still serious, diseases (eg, cardiovascular-affected patients).

Moreover, considering that the average survival time for patients with COVID-19 that die has been measured to be approximately 10 days after ICU admission, the need to plan resources is urgent. It may involve making new ICU beds and planning logistics to move patients among regions or to optimize the grouping of patients with COVID-19 in dedicated health structures. It is trivial that such a decision must be based on the correct estimation of ICU beds that are occupied by patients, but this estimation is still a matter of discussion [26].

### Model Description and Assessment

We report here the description and assessment of the proposed model by using Italian cases.

We start by considering a time window of six consecutive infection values (one reading per day) from the official Italian COVID-19 data set. We then calculate an exponential fitting function for these values, since we know that the viral phase follows an exponential growth.

**Figure 1:**
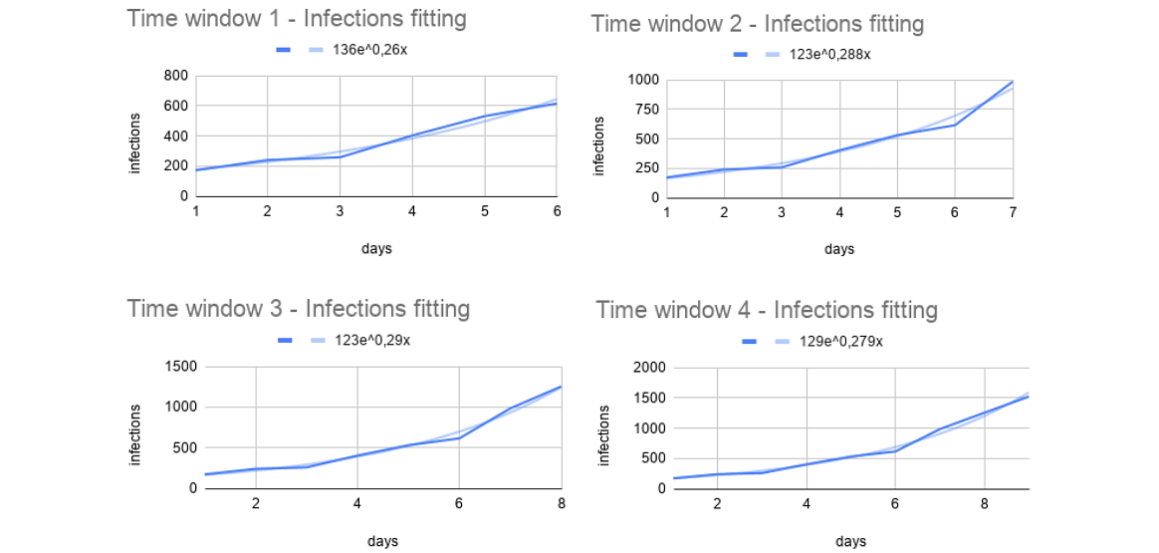
Exponential fitting of infection levels for the first four time windows. Each is longer than the previous by 1 day. The shown time windows are W1 (6 days), W2 (7 days), W3 (8 days), and W4 (9 days).

In Figure 1, the first four time windows (ie, 6, 7, 8, and 9 days) and the related fitting functions are reported. The exponential fitting function for the first window is 
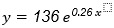
, where *x* is time (ie, days) and *y* is the number of infections. We use the calculated fitting equation to predict the number of patients infected with COVID-19 for the succeeding days; for the first time window, we predicted 1086 total infections on the seventh day. We compared the predicted value with the observed infections (ie, real number of infections) from the data set, and we calculated the difference and the percentage increase, which will be useful during the assessment phase. We then proceeded by extending the time window by 1 day, and we redid all the steps (exponential fitting, prediction of the infections for the succeeding day, difference, and percentage increase).

**Figure 2:**
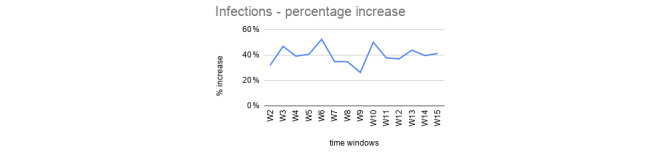
Percentage increase between the predicted and the observed number of infections. On the x-axis, we have time windows and, on the y-axis, the percentage increase of the predicted infection values with respect to the observed ones.

To assess the precision of the first step, we considered the calculated percentage increase between the predicted values and the observed ones. As reported in Figure 2, the percentage increase was around 40%.

**Figure 3:**
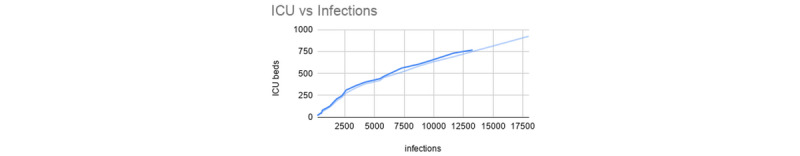
Correlation between occupied ICU beds and COVID-19 infections. ICU: intensive care unit.

In the second step, we consider the number of occupied ICU beds as a function of the number of COVID-19 infections. In this case, we adopted the weighted average as a fitting function. Figure 3 depicts this correlation (in blue) between total infections (x-axis) and ICU beds occupied (y-axis) together with the weighted average fitting for the whole data set (in light blue).

**Figure 4:**
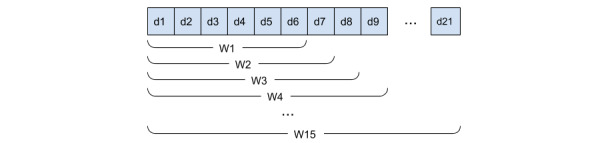
Time windows W1, W2, …, W15 are defined incrementally starting from a period of 6 consecutive days, which has been considered the minimum number of data points to calculate the fitting function.

**Figure 5:**
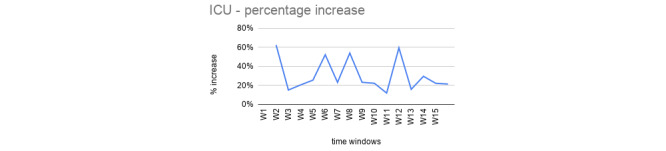
Percentage increase between the predicted and the observed ICU beds occupied. ICU: intensive care unit.

For each time window (W1, W2, …, W15; see Figure 4), we consider three adjacent data point coordinates (infections for the x-axes and ICU for the y-axes) to calculate a linear equation as a weighted average fitting function for the values contained in it. We then used such a function to estimate the future ICU bed occupation for the following day by using the predicted infected value. We then calculated the difference between the predicted and the observed ICU values, and similarly to the first step, we reported the percentage increase between the two. Table 2 reports an example of percentage increase values of the predicted versus observed ICU resources for an Italian region’s data set. The percentage increase is above 40% for only a few values, but the majority are near 20%, as represented in Figure 5.

**Table 2:** Observed versus predicted ICU beds for the Lombardia Region for each time window (W1, ..., W15) considered in the time period from February 24 to March 15, 2020.

Observed ICU^a^ beds^b^, n	Predicted ICU beds^c^, n	Percentage increase (%)
106	172	62
127	146	15
167	201	20
209	262	25
244	371	52
309	380	23
359	552	54
399	491	23
440	537	22
466	521	12
560	892	59
605	700	16
650	841	29
732	893	22
767	930	21

^a^ICU: intensive care unit.

^b^Observed (ie, real) ICU beds measured during the COVID-19 emergency.

^c^Calculated by our model.

We applied the described method both at the national and regional scale, and we report the results in the next section.

## Results

In this section, we show the application of the described model to the Italian official COVID-19 data set [27], and we briefly discuss the limitations of the current model and its application to the Italian use case at a regional level. We show that, by using our method, it has been possible to predict future ICU bed occupancy with fair accuracy.

The proposed model works in the exponential phase of the infection spread, while for the nonexponential stage, other models can be used such as the Verhulst logistic model [9]. In its present form, the model is tailored to the Italian COVID-19 data set. However, with minimal adaptation, it could work with other data sets of infectious diseases with different data schemas.

The presented model works in the exponential phase of the infection. Model sensitivity has not been considered in the case of this model because we focused only on the exponential growth of the infections, which is the crucial moment that ICU bed and resource availability are most stressed and inadequate.

Changing the assumption (ie, nonexponential growth) is considering a time window in which ICU resources are surely available with respect to the requests. For instance, we performed experiments that modeled the nonexponential infection phase with a Verhulst logistic model, but again, when there is no emergency, the ICU predictions are not useful since resources are largely available.

The final goal is to predict the number of future beds needed in the ICUs as a function of the level of infection in a given region. The ability to predict future resource occupation can be a powerful and useful tool for local decision makers with the responsibility of managing and optimizing clinical resources during the emergency.

We report the application of the presented model for three Italian regions: Lombardia, Toscana, and Sicilia, which represent a balanced sample of northern, central, and southern Italian regions. We extracted and transformed the relevant data from the official Italian COVID-19 data set and considered the total number of patients who were infected and the ICU beds occupied by patients with COVID-19 reported by the various local health structures.

In the data set, we have 17 total features (eg, latitude, longitude, date, the total number of infections, number of patients who are hospitalized, number of deaths, and number of recoveries) and one reading per day for each region. We selected the features of interest and aggregated the tuples by region before estimating the prediction model parameters. For each region, we considered the number of infections, and we calculated an exponential fitting equation. By using such an equation, we were able to estimate the number of patients who will be infected in the succeeding days. We then considered the relation between ICUs and infections, with which we can use the predicted infection levels to estimate future values of ICU and resource occupation. These predictions can be a valuable tool for rapidly planning ICU resources in case of shortages during clinical emergencies in general, for instance, by reallocating patients in other regions with lower levels of ICU occupancies.

**Figure 6:**
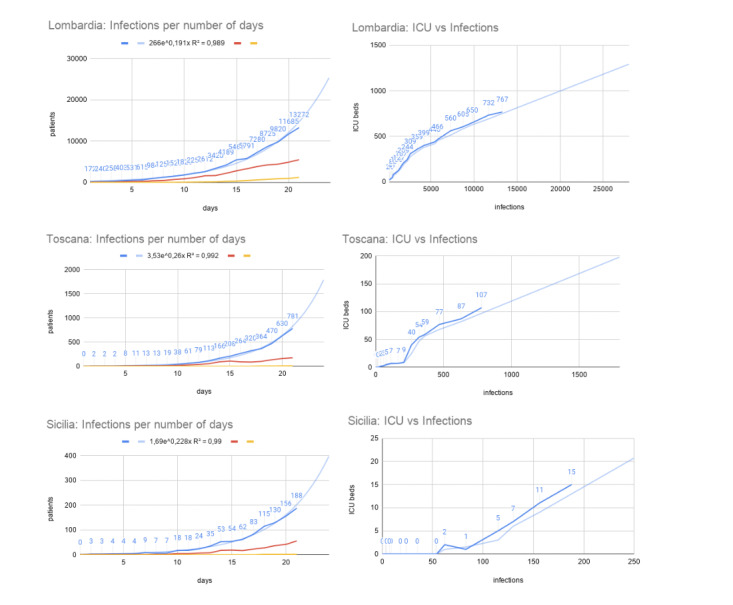
The figure depicts Lombardia, Toscana, and Sicilia regions as representative of infection situations in the northern, central, and southern regions of Italy, respectively, in a time window between days (0 days to 21 days; ie, February 24 to March 15, 2020) of infection in the official data set (ie, 3 weeks). In the left column, we report the number of infections per day, while in the right column, we have the occupied ICU beds as a function of the number of COVID-19 infections. In light blue, we report the fitting functions for the considered data (left column: exponential function; right column: weighted average with a modulus of 3). The red lines in the left column show the hospitalized patients with symptoms, and the yellow lines show the number of deaths. ICU: intensive care unit.

In Figure 6 (upper left), we report COVID-19 data about the Lombardia region between February 24 and March 15, 2020, a time period in which infection levels (in blue) were growing in an exponential fashion. We report the exponential fitting function for the infections (in light blue), the number of hospitalized with symptoms (in red), and the number of deaths related to COVID-19 (in yellow). Figure 6 (upper right) depicts the occupied ICU beds as a function of the number of people infected with COVID-19 in the same aforementioned time period. The fitting function (in light blue) is a weighted average with a modulus of 3, with which we predict future ICU bed occupation from a given infection value.

Similar to the Lombardia region, we report the application of the proposed model to COVID-19 infection data and resource necessity prediction for both the Toscana and Sicilia regions (central and lower part of Figure 6, respectively). We report three example regions (Figure 6) to represent the northern regions (ie, Lombardia; which are the more affected part of the country), the central regions (ie, Toscana), and the south (ie, Sicilia). They are needed to show how COVID-19 diffused differently from the north to the south of Italy.

## Discussion

### Limitations

The presented model only works in a scenario of an exponential growth of infections, since it is a crucial moment in which ICU bed and resource availability are most stressed and inadequate.

The nonexponential phase might be modeled, for instance, by using models such as the Verhulst logistic one. However, our focus is on time windows in which resources are scarcely available with respect to the rapidly increasing requests, such as ICU beds in the COVID-19 pandemic.

Another limitation of the proposed model regards the impossibility of considering predictions too far ahead in the future, limiting the applicability of the prediction to a few days. However, this is generally sufficient to help in planning ICU resources during an emergency.

### Comparison With Prior Work

Modeling of the COVID-19 spread is currently a hot topic considering the pandemic. Consequently, many different works have been proposed. Some of them are based on a deterministic model that uses ordinary differential equations for predicting the number of infected people (eg, [11,12,28]). Some other approaches use Markov modeling and compartmental models (eg, [29-31]). To the best of our knowledge, only the work of Rossman et al [10] presents a scalable granularity (at a state level). With respect to those works, we also tried to predict ICU needs (including data about existing ICU occupancy and the trend of ICU use) with the aim to support health care managers.

### Conclusion

The COVID-19 pandemic has been characterized by the rapid spread of an aggressive virus, which has stressed the health system. We think that patient management is strictly related to the ability of health structures to deal with this kind of disease, which requires nonstandard protocols such as the use of respiratory devices. We think that, by using a scalable predictive model at regional and district levels, the granularities may support decision makers (eg, national governments) in better managing the emergency.

The COVID-19 pandemic has reached different regions in various countries worldwide. Furthermore, it is expected that the virus will cyclically reappear in the near future. To this end, the proposed model could be applied during these new outbreaks and as a decision support tool in other similar pandemics or situations where resource prediction is necessary.

## Abbreviations

ICU: intensive care unit
